# Static and dynamic interactions within the triple-network model in stroke patients with multidomain cognitive impairments

**DOI:** 10.1016/j.nicl.2024.103655

**Published:** 2024-08-10

**Authors:** Yingying Wang, Hongxu Chen, Caihong Wang, Jingchun Liu, Peifang Miao, Ying Wei, Luobing Wu, Xin Wang, Peipei Wang, Yong Zhang, Jingliang Cheng, Siyuan Fan, Guifang Sun

**Affiliations:** a Department of MRI, Henan Key Laboratory of Magnetic Resonance Function and Molecular Imaging, The First Affiliated Hospital of Zhengzhou University, Zhengzhou, China; b Huaxi MR Research Center (HMRRC), Functional and Molecular Imaging Key Laboratory of Sichuan Province, Department of Radiology, West China Hospital, Sichuan University, Chengdu, China; c Cardiff University Brain Research Imaging Centre, United Kingdom; d Department of Radiology, Tianjin Key Laboratory of Functional Imaging, Tianjin Medical University General Hospital, Tianjin, China; e Cardiovascular Center, Liyuan Hospital, Tongji Medical College, Huazhong University of Science and Technology, Wuhan, China; f Clinic Center of Human Gene Research, Union Hospital, Tongji Medical College, Huazhong University of Science and Technology, Wuhan, China; g Department of Neurology, The First Affiliated Hospital of Zhengzhou University, Henan Province 450052, China

**Keywords:** Resting-state fMRI, Stroke, Static functional network connectivity, Dynamic functional network connectivity, Dynamic community structure

## Abstract

**Background:**

Internal capsule strokes often result in multidomain cognitive impairments across memory, attention, and executive function, typically due to disruptions in brain network connectivity. Our study examines these impairments by analyzing interactions within the triple-network model, focusing on both static and dynamic aspects.

**Methods:**

We collected resting-state fMRI data from 62 left (CI_L) and 56 right (CI_R) internal capsule stroke patients, along with 57 healthy controls (HC). Using independent component analysis to extract the default mode (DMN), executive control (ECN), and salience networks (SAN), we conducted static and dynamic functional network connectivity analyses (DFNC) to identify differences between stroke patients and controls. For DFNC, we used k-means clustering to focus on temporal properties and multilayer network analysis to examine integration and modularity Q, where integration represents dynamic interactions between networks, and modularity Q measures how well the network is divided into distinct modules. We then calculated the correlations between SFNC/DFNC properties with significant inter-group differences and cognitive scales.

**Results:**

Compared to HC, both CI_L and CI_R patients showed increased static FCs between SAN and DMN and decreased dynamic interactions between ECN and other networks. CI_R patients also had heightened static FCs between SAN and ECN and maintained a state with strongly positive FNCs across all networks in the triple-network model. Additionally, CI_R patients displayed decreased modularity Q.

**Conclusion:**

These findings highlight that stroke can result in the disruption of static and dynamic interactions in the triple network model, aiding our understanding of the neuropathological basis for multidomain cognitive deficits after internal capsule stroke.

## Highlights

• Strokes disrupt static and dynamic interactions in the triple-network model.
• Functional network connectivity exhibits lesion-side effects in stroke patients.
• Altered functional connectivity correlates with cognitive performance.

## 1 Introduction

The internal capsule, deeply situated in the brain, comprises numerous white matter fiber tracts connecting the thalamus, basal ganglia, cerebral cortex, and brainstem (Safadi, 2018). This structure's disruption can lead to motor control issues, sensory dysregulation, and multidomain cognitive impairments (Wang, 2022), such as memory, attention, and executive function. In neuroscience, internal capsule strokes are increasingly viewed as disorders of brain network connectivity (Wang, 2019), impacting not just a single area but the broader interactions across multiple brain networks.

Resting-state functional magnetic resonance imaging (rs-fMRI), as a valuable and non-invasive tool, has been extensively used to quantify intrinsic functional networks connectivity in many neuropsychiatric diseases (Tang, 2021). Recent advances in mapping intrinsic brain connectivity networks have provided crucial insights into the mechanisms underlying various aspects of human behavior. For instance, research has demonstrated that network localization can elucidate the neurobiological mechanisms of suicide by identifying key brain networks, such as the default mode and anterior salience networks (Zhang et al., 2024). Similarly, studies on auditory verbal hallucinations (AVH) in schizophrenia have shown that AVH-state and AVH-trait brain alterations localize to distinct networks, with AVH-state involving auditory and salience networks, and AVH-trait involving the caudate and inferior frontal gyrus (Mo, 2024). Additionally, research on healthy individuals has shown that brain connectivity within and between large-scale networks is linked to behaviors such as sleep quality, working memory, and attention (Cai, 2021). Of the many intrinsic brain networks, the triple-network model proposed by Menon (Menon, 2011), including the default-mode network (DMN), the executive control network (ECN), and salience network (SAN), emphasizes the cooperation among networks. Specifically, the DMN, mainly consists of the ventromedial prefrontal cortex (vmPFC) and posterior cingulate cortex (PCC), is associated with internal thought and autobiographical memory (Buckner et al., 2008). The ECN, primarily includes in the lateral posterior parietal cortex and dorsolateral prefrontal cortex, is involved in multiple cognitive processes, such as working memory, decision-making, and attention (Liang et al., 2016). The SAN, primarily composed of the dorsal anterior cingulate cortex (dACC) and the bilateral anterior insula (AI), has numerous structural and functional connections (FCs) with many brain regions of DMN and ECN (Averbeck and Seo, 2008). SAN is responsible for processing salience information and regulating the balance of cognitive resources between different large-scale networks such as DMN and ECN, to facilitate goal-directed behavior (Menon, 2011). It has been demonstrated that triple-network model provides a common framework for understanding the dysfunction in core neurocognitive networks across multiple psychiatric and neurological disorders, such as obsessive–compulsive disorder (Fan, 2017); schizophrenia (Jiang, 2017) and attention-deficit/hyperactivity disorder (Gao, 2019). Further study of triple-network model alterations could help us better understand the pathological mechanisms of multidomain cognitive impairments after a stroke.

Traditional studies of functional network connectivity (FNC) have indeed offered valuable insights into the neurobiological foundations of stroke, but they have primarily focused on static functional network connectivity (SFNC) patterns. In fact, the brain operates as a dynamic system, undergoing shifts in connectivity over scanning time (Allen, 2014). These dynamic reconfigurations are essential for effective information transmission, cognitive adaptability, and rapid reactions to the external environment (Baker, 2014). Recently, researchers have found that the dynamic functional network connectivity (DFNC) method can uncover certain characteristics that are overlooked in SFNC (Nomi, 2016). The properties identified by DFNC may be associated with cognitive impairment (Yue, 2023); motor injury (Wang, 2023), aphasia (Xu, 2022), post-stroke depression (Yao, 2020) and spatial neglect (Spadone, 2023) in stroke patients; in addition, it might be regarded as a potential marker to predict the acute function impairment and chronic recovery (Bonkhoff, 2021) in stroke patients. Therefore, a combination of the SFNC and DFNC approaches, can help us gain a comprehensive understanding of the static and dynamic interactions across different brain networks within triple-network model in stroke patients with multidomain cognitive impairments.

Conventional DFNC analyses, like k-means clustering, group similar time windows based on connectivity patterns, providing a snapshot of dynamic brain states. However, k-means clustering treats each time window independently, ignoring the temporal continuity and interdependencies between consecutive windows. To address this limitation, we employed a multilayer network approach, where an adjacency matrix was estimated for each time window, and all windows were interconnected into a multilayer network, with each time window as a layer (Yang, 2021). This method constructs a supra-adjacency matrix, capturing both intra- and inter-layer connections, thus providing a comprehensive view of the dynamic interactions. The multilayer network approach allows us to investigate the dynamic community structure of brain networks, where groups of nodes exhibit stronger connections among themselves and weaker connections with nodes outside their communities (Sporns and Betzel, 2016). Over time, nodes in several predefined functional networks may be in the same community at one moment and then be in the same community with nodes from other predefined functional networks at the next moment. To detect these communities, we used the GenLouvain community detection algorithm. The consistency of community assignment between nodes is characterized with a module allegiance matrix, where each element gives the relative frequency that one node shares the same community with another across time windows (Chai et al., 2016). The integration coefficient, calculated from the module allegiance matrix, reflects the probability of a node being in the same community as nodes from other networks, indicating dynamic interactions between functional networks. Recent studies have shown that dynamic community structure is linked to neurophysiological processes like working memory (Finc, 2020), attention (Shine et al., 2016), and reinforcement learning (Gerraty, 2018). Furthermore, the properties of dynamic community structure may serve as potential biomarkers for diseases such as schizophrenia (Gifford, 2020), depression (Han, 2020) and attention-deficit/hyperactivity disorder (Cui, 2021).

Cerebral hemispheric asymmetry can result in differential reorganization in the homologous regions of the left and right hemispheres following a stroke (Corballis, 2014). Due to right-handedness, the left hemisphere is typically dominant in the majority of adults. Consequently, strokes in the dominant or non-dominant hemispheres can lead to varying degrees of behavioral impairment, along with diverse damage and reorganization in brain structure and function. Empirical evidence highlights the distinct impact of lesion side on brain properties. Right-hemisphere lesions are associated with significant increases in gray matter volume (GMV) in regions such as the bilateral paracentral lobule and supplementary motor area (SMA) (Diao, 2017), as well as significant decreases in voxel-mirrored homotopic connectivity (VMHC) in the bilateral lingual gyrus and left precuneus (Wu, 2021), and reduced dynamic regional homogeneity (dReHo) in the middle cingulate cortex (Wang, 2023), changes not observed in left-hemisphere lesions. In addition, left-sided stroke patients exhibit more extensive disruptions in structural connectivity (Wang, 2019) and greater aberrant temporal dynamics of intrinsic brain activity (Wang, 2023) compared to right-sided stroke patients. However, there remains a gap in comprehensively understanding the spatiotemporal patterns of FNC within the triple-network model in patients with infarctions on different hemispheric sides.

**Fig. 1:**
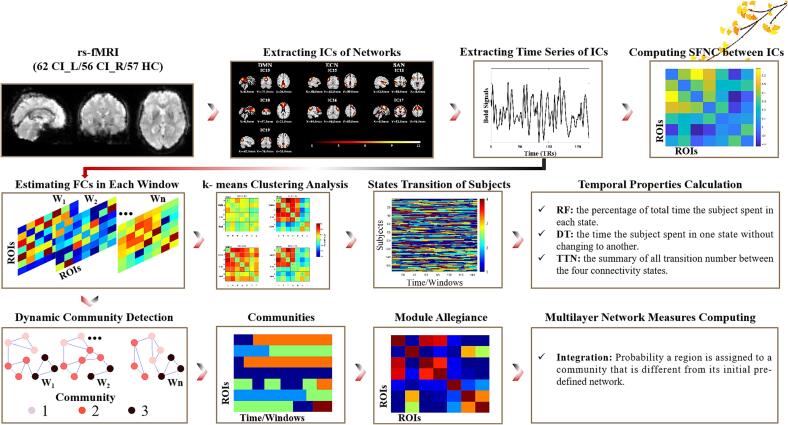
Flowchart summarizing the major steps of the current analytical framework. Abbreviations: CI_L=patients with infarct in left-sided internal capsule; CI_R=patients with infarct in right-sided internal capsule; HC=healthy controls; ICs = independent components; SFNC=static functional network connectivity; RF=reoccurrence fraction; DT=dwell time; TTN=total number of transitions.

In this study, we aimed to explore the characteristics of SFNC and DFNC (including the temporal properties and dynamic community structure) based on the triple-network model. Given that patients with different lesion sides may exhibit distinct patterns of functional connectivity changes, we subdivided stroke patients into two groups (patients with left and right internal capsule stoke, referred to as CI_L and CI_R, respectively) based on the side of the lesions. The pipeline of the analysis strategy for this study is displayed in Fig. 1. We hypothesized: (a) stroke patients exhibit disrupted SFNC and DFNC patterns in triple-network model; (b) these abnormalities are related to multidomain cognitive impairment in stroke patients; (c) the alterations of SFNC and DFNC display lesion-side effect in stroke patients. This study may improve our understanding of the neurophysiological processes based on the triple-network model in stroke patient.

## 2 Methods

### 2.1 Participants

This research was carried out at the First Affiliated Hospital of Zhengzhou University and Tianjin Medical University General Hospital. The study received approval from the ethical committees of the local hospitals, and all participants provided informed consent. The inclusion/ exclusion criteria for stroke and HC groups were displayed in the **Part 1** of Supplementary Materials. After data quality control, a total of 62 patients with CI_L (40 males and 22 females, age 55.21 ± 8.04 years), 56 patients with CI_R (36 males and 20 females, age 54.02 ± 9.07 years), and 57 randomly selected HC participants (34 males and 23 females, age 55.81 ± 7.98 years) were enrolled in the study.

### 2.2 Behavioral measures

In this study, cognitive tasks were performed using E-Prime 2.0 software (https://pstnet.com/products/e-prime/). Visual attention was evaluated with the Flanker task (FT) (Eriksen and Eriksen, 1974), involving arrow stimuli. Working and spatial memory were tested with modified versions of the number back (NBT) (Stollstorff, 2010) and spatial back tasks (SBT) (Vuontela, 2003). Above cognitive performance was determined by reaction times (RT) and accuracy for correct responses. Episodic memory was measured using the Rey Auditory Verbal Learning Test (RAVLT) (Allen, 2018), including short-term (RAVLT_S) and long-term memory scores (RAVLT_L). Executive function was assessed using the Trail Making Test (TMT) (Llinàs-Reglà, 2017), which includes two parts (TMT-A and TMT-B). Additionally, motor function was evaluated with the Fugl-Meyer test, focusing on whole extremity and upper limb movements. For detailed steps, please see the **Part 2** of Supplementary Materials**.**

### 2.3 Data acquisition

At both medical centers, MR images of all subjects were acquired using the same parameters on two scanners with the same type (Discovery MR750 3.0 Tesla, General Electric, Milwaukee, WI, USA). During the scanning process, all participants were asked to close their eyes and remain motionless. The detail parameters of MR images were displayed in the **Part 3** of Supplementary Materials.

### 2.4 Data preprocessing

**Fig. 2:**
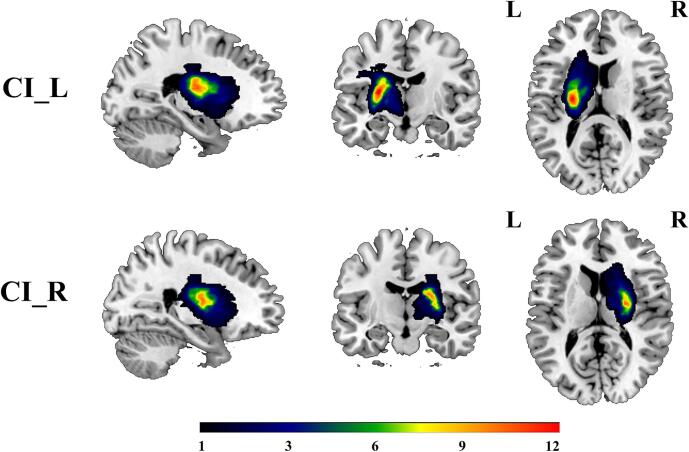
The overlapped lesion maps of CI_L and CI_R patients. The color bar indicates the number of subjects with lesions in each voxel. Abbreviations: CI_L=patients with infarct in left-sided internal capsule; CI_R=patients with infarct in right-sided internal capsule; L=left hemisphere, R=right hemisphere.

Fig. 2 depicted the probability map of the lesion location. The three-dimensional T1-weighted images (3D-T1WI) were first normalized to the standard Montreal Neurological Institute (MNI) space. The lesions were then artificially delineated layer by layer on the normalized 3D-T1WI by an experienced neuroradiologist using the MRIcron software (https://www.mccauslandcenter.sc.edu/mricro/mricron/), resulting in individual lesion masks for each stroke patient. An average lesion mask was generated by overlapping these individual masks. Finally, the average lesion mask was superimposed onto the standard MNI template. The classification of stroke patients into these groups is based on the lateralization of lesions observed in 3D T1-weighted MRI images. Specifically, CI_L consists of patients with stroke lesions located in the left internal capsule, and CI_R includes those with lesions in the right internal capsule. The Data Processing & Analysis for Brain Imaging (DPABI; https://rfmri.org/DPABI) program was used to preprocess all MRI data. The detail preprocessing steps were presented in the **Part 4** of Supplementary Materials. To minimize head motion effects on SFNC and DFNC analyses, participants were excluded if they had a maximum displacement over 2.0 mm, a maximum rotation above 2.0 degrees, or mean framewise displacement (FD) (Power et al., 2012) exceeding 0.5. Statistical comparisons showed no significant differences in mean FD between the CI_L and HC groups (*p* = 0.056), between the CI_R and HC groups (*p* = 0.081), or between the CI_L and CI_R groups (*p* = 0.977). The detail processes of controlling for head motion were displayed in the **Part 5** of Supplementary Material.

### 2.5 Identification of resting-state networks

Using the Group ICA of fMRI Toolbox software (GIFT version 4.0b; https://icatb.sourceforge.net), RSNs were identified using Spatial Independent Component Analysis. Firstly, it involved data reduction through principal component analysis (PCA). Then, the infomax algorithm was used to extract independent spatial maps and time courses for each component. Finally, back-reconstruction was applied to obtain individual-level components. Above steps were performed 100 times and the number of independent components were auto estimated as 23. Finally, seven independent components (ICs) belong to the three RSNs were extracted by ICA, including the default mode network (DMN, IC13: posterior cingulate cortex (PCC); IC18: medial prefrontal cortex (MPFC); IC19: inferior parietal lobule (IPL)), the left and right executive control networks (ECN, IC25: LECN; IC26: RECN), and the salience network (SAN, IC12: insula; IC17: dorsal anterior cingulate cortex (dACC)). The components and locations of these RSNs were consistent with previous studies (Smith, 2009), and the details of the spatial distribution of the three networks are presented in Figure S1, detail information see the Supplementary Material.

### 2.6 Post-processing of time courses

Following previous studies, we performed additional post-processing steps on time courses of the RSNs, including (a) removing linear, quadratic, and cubic detrends, (b) regressing out six realignment parameters and their temporal derivatives (in x-, y-, and z-direction as well as pitch, roll, and yaw), (c) low-pass filtering of 0.15 Hz, and (d) removing spikes to ensure that the signal analysis is not affected by artifactual spikes (Tu, 2019). Finally, the residual time courses were used to perform SFNC and DFNC analyses.

### 2.7 Static functional network connectivity analysis

Pearson correlation coefficients (*r*) were computed between the time courses of seven ICs belonging to triple-network model in the CI_L, CI_R, and HC groups to establish SFNC. This process yielded a connectivity matrix of dimensions C×C for each subject (where C represents the number of identified ICs). To enhance normality, the *r*-values were transformed into *z*-values using Fisher's *r*-to-*z* conversion.

### 2.8 K-means clustering analysis of DFNC

#### 2.8.1 DFNC matrix computation

The DFNC toolbox of GIFT was used to perform DFNC analysis using a sliding-window technique. We chose the length (22TRs) with a Gaussian of σ = 3 TRs, in a step of 1 TR, resulting in 148 windows, based on previous researches (Allen, 2014, Damaraju, 2014). Thus, for each subject and each window, we obtained a functional connectivity matrix reflecting the time-varying FCs between seven ICs belonging to triple-network model. Finally, to increase the normality of the correlation distribution, a Fisher's *r*-to-*z* transformation was applied to all DFNC matrices, where *r* is the Pearson correlation coefficient between the time series of ICs.

#### 2.8.2 K-means clustering

**Fig. 3:**
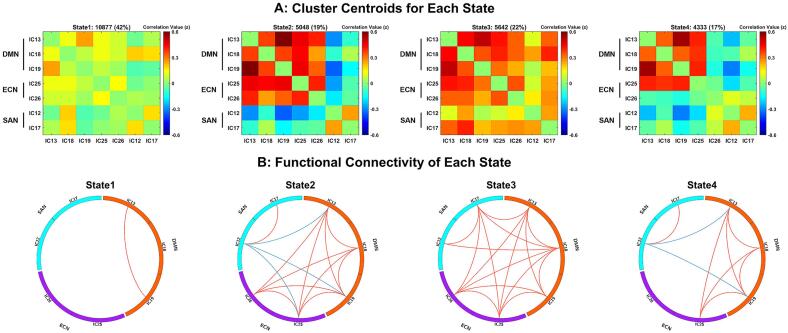
A: The centroid of each functional network connectivity state with the total number of occurrences and percentage of total occurrences. B: Functional connectivity of each state, representing the functional connectivity with the absolute values of connection strengths greater than the average absolute value of connection matrix strengths across all four states. Abbreviations: DMN=default mode network; ECN=execution control network; SAN=salience network.

K-means clustering was employed to determine specific DFNC patterns of the windowed covariance matrices, and the squared Euclidean distance (500 iterates and 150 replicates) was used to evaluate the similarity between different time windows (Malhi et al., 2019). Using the elbow criterion computed as the ratio between within-cluster distance to between-cluster distances (Allen, 2014), the optimal number of clusters was estimated to be four (k = 4), with the centroids of four clusters illustrated in Fig. 3.

#### 2.8.3 Dynamic properties analysis

We analyzed the temporal features of DFNC states in the following ways: (1) reoccurrence fraction (RF, the percentage of total time the subject spent in each state); (2) dwell time (DT, the time the subject spent in one state without changing to another); (3) total number of transitions (TTN, the summary of all transition number between the four connectivity states).

### 2.9 Multilayer network analysis of DFNC

#### 2.9.1 Multilayer network construction

In this method, we constructed an adjacency matrix for each time window, interconnecting all 148 windows into a multilayer network. In this structure, each independent component (IC) is represented as a node, with Pearson's correlations between IC pairs depicted as edges, and each time window forming a layer. We focused on the connections between the same ICs across adjacent layers. This multilayer network can be simplified using a supra-adjacency matrix (Cozzo and Moreno, 2016). For detailed expressions and explanations of the supra-adjacency matrix, please refer to the **Part 6** of Supplementary Materials.

#### 2.9.2 Multilayer community detection

To explore the temporal evolution of community in the multilayer network, we employed the GenLouvain community detection algorithm (Jutla et al., 2011), rooted in the concept of the Louvain-like greedy algorithm. Modularity serves as a formal metric for investigating the optimal assignment of these communities. In essence, this algorithm determines the community membership for each IC by maximizing the multilayer modularity quality function Q, which is defined as:

$$Q=\frac{1}{2\mu }\sum_{\mathrm{ijlr}}\left\{\left(A_{\mathrm{ijl}}-\gamma_{l}P_{\mathrm{ijl}}\right)\delta_{\mathrm{lr}}+\delta_{\mathrm{ij}}\omega_{\mathrm{jlr}}\right\}\delta \left(g_{\mathrm{il}},g_{\mathrm{jr}}\right)$$

The detailed explanation of the modularity quality function Q is provided in **Part 6** of the Supplementary Materials. For each time window (layer), we identified IC community partitions by optimizing the multilayer modularity function Q. Given the inherent randomness of the Louvain-like greedy algorithm, the outputs vary slightly between runs. Consequently, we ran the algorithm 50 times to obtain averaged final values for the quantitative indicators.

#### 2.9.3 Multilayer network properties analysis

**Module Allegiance:** Module allegiance characterizes the consistency of community assignment between two ICs during the scan. An N×N square matrix (N is the number of ICs) was formed to create module allegiance matrix for each participant, in which each element *P_ij_* gives the relative frequency that one IC*_i_* shares the same community with another IC*_j_* across time windows (Chai et al., 2016). The detailed formula and explanation are provided in **Part 6** of the Supplementary Materials. The module allegiance between two RSNs is calculated by averaging the *P_ij_* values of all ICs between the two RSNs.

**Integration:** The integration coefficient (Cui, 2021), calculated from the module allegiance matrix, quantifies the dynamic interactions between ICs within a network and ICs from other networks (Wu et al., 2023). These interactions involve reconfiguration over time, where ICs in the same community at one time point may shift to join communities with ICs from other networks at the next time point. A higher integration coefficient indicates more frequent interactions with ICs from different networks across time windows, reflecting greater temporal flexibility and adaptive capacity. The detailed formula and explanation are provided in **Part 6** of the Supplementary Materials. The RSN-level integration coefficient was obtained by averaging IC-level integration across each RSN; the RSN-RSN integration is represented by the module allegiance matrix between RSNs.

### 2.10 Statistical analysis

#### 2.10.1 Comparison of demographic and behavioral scales between groups

We compared differences in demographic information and behavioral scales between the CI_L and HC groups, between the CI_R and HC groups, as well as between the CI_L and CI_R groups. The two-sample t-tests were used to explore group differences of the age, years of education and behavioral scales. A chi-square test was performed to explore group differences of gender. The threshold for statistical significance was set to *p* < 0.05, uncorrected.

#### 2.10.2 Comparison of SFNC/DFNC properties between groups

We used a general linear model to detect differences in SFNC and DFNC properties separately between the CI_L and HC groups (CI_L analysis), and between the CI_R and HC groups (CI_R analysis). Age, gender, years of education, and mean framewise displacement (FD) were included as covariates in the model. The adjusted level of significance for all these tests was set at *p* < 0.05, FDR corrected. For SFNC, we focused on the functional connectivities between each pair of ICs, comparing them and applying FDR correction to the resulting *p*-values.

Regarding DFNC, it can be divided into two parts: k-means cluster analysis and multilayer network analysis. In the k-means cluster analysis, we computed three temporal properties: RF, DT, and TTN. Specifically, the RF and DT of the four states were compared between groups, resulting in four *p*-values for each parameter, which were then subjected to FDR correction. The TTN value, being a unique single value for each participant, was not subjected to FDR correction.

In the multilayer network analysis, we calculated the following parameters: integration parameters at the IC- and RSN-levels; the RSN-RSN integration (RSN-level module allegiance); as well as the quality of community (quantified by the modularity Q of the multilayer network). This resulted in 7 *p*-values for IC-level integration, 3 *p*-values for RSN-level integration, and 3 *p*-values for RSN-RSN integration, all of which were FDR-corrected. The modularity Q value, being a unique single value for each participant, was not subjected to FDR correction.

#### 2.10.3 Brain-behavioral relationship

We calculated the Pearson's correlation with the clinical variables in the CI_L, CI_R and HC groups (*p* < 0.05, uncorrected) for each SFNC and DFNC measure that showed significant intergroup differences (CI_L vs HC, CI_R vs HC). The FT, RAVLT, NBT, SBT, TMT and FMT were among the clinical variables.

## 3 Results

### 3.1 Demographic and behavioral parameters

This study included 62 CI_L, 56 CI_R patients and 57 HC. No significant inter-group difference was observed in age (for CI_L vs. HC, *p* = 0.688; for CI_R vs. HC, *p* = 0.273; for CI_L vs. CI_R, *p* = 0.457), years of education (for CI_L vs. HC, *p* = 0.407; for CI_R vs. HC, *p* = 0.226; for CI_L vs. CI_R, *p* = 0.676), gender (for CI_L vs. HC, *p* = 0.588; for CI_R vs. HC, *p* = 0.616; for CI_L vs. CI_R, *p* = 0.979), lesion size (for CI_L vs. CI_R, *p* = 0.707) and duration (for CI_L vs. CI_R, *p* = 0.127).

As for the behavioral assessment scores, compared to HC subjects, CI_L patients displayed worse performance in visual attention (for FT_Accuracy, *p* = 0.003), working memory (for NBT_Accuracy, *p* = 0.001), spatial memory (for SBT_Accuracy, *p* = 0.001; for SBT_RT, *p* = 0.043) and executive function (for TMT_A, *p* = 0.047; for TMT_B, *p* = 0.000); CI_R patients displayed worse performance in visual attention (for FT_Accuracy, *p* = 0.000), working memory (for NBT_Accuracy, *p* = 0.000) and spatial memory (for SBT_Accuracy, *p* = 0.005). The detail demographic and clinical data of CI_L, CI_R and HC subjects are described in Table 1. The distribution of scores from the cognitive function assessments is presented in Figure S2 of the Supplementary Material.

**Table 1:** Demographic and clinical data of stroke patients and healthy controls.

**Variables**	**Mean ± Std**			**CI_L vs. HC**		**CI_R vs. HC**		**CI_L vs. CI_R**	
	**CI_L (n = 62)**	**CI_R (n = 56)**	**HC (n = 57)**	***x^2^/t***	***p***	***x^2^/t***	***p***	***x^2^/t***	***p***
**Age (Years)**	55.21 ± 8.04	54.02 ± 9.07	55.81 ± 7.98	−0.403	0.688	−1.103	0.273	0.746	0.457
**Gender** **(Male/Female)**	40/22	36/20	34/23	0.295	0.588	0.254	0.616	0.001	0.979
**Education (Years)**	9.65 ± 3.49	9.36 ± 3.86	10.11 ± 2.43	−0.833	0.407	−1.220	0.226	0.420	0.676
**Mean FD**	0.17 ± 0.10	0.17 ± 0.12	0.14 ± 0.07	1.929	0.056	1.762	0.081	−0.029	0.977
**Size (cm^3^)**	0.73 ± 0.79	0.67 ± 0.81	/	/	/	/	/	0.377	0.707
**Duration (Days)**	459.45 ± 404.54	358.98 ± 295.59	/	/	/	/	/	1.537	0.127
**FMT_Upper**	59.63 ± 11.80	59.59 ± 12.57	/	/	/	/	/	0.018	0.986
**FMT_Whole**	90.66 ± 15.65	90.50 ± 16.60	/	/	/	/	/	0.054	0.957
**FT_Accuracy**	0.94 ± 0.06	0.93 ± 0.08	0.97 ± 0.05	−3.079	**0.003**	−3.644	**0.000**	0.963	0.338
**FT_RT**	629.20 ± 224.89	655.48 ± 217.43	612.91 ± 201.25	0.414	0.680	1.070	0.287	−0.640	0.524
**RAVLT_S**	45.05 ± 11.45	49.09 ± 12.72	48.47 ± 8.19	−1.872	0.064	0.303	0.763	−1.791	0.076
**RAVLT_L**	10.53 ± 2.68	11.36 ± 2.65	11.07 ± 2.85	−1.050	0.296	0.550	0.583	−1.665	0.099
**NBT_Accuracy**	0.88 ± 0.12	0.88 ± 0.09	0.94 ± 0.04	−3.331	**0.001**	−4.006	**0.000**	−0.026	0.979
**NBT_RT**	855.00 ± 272.99	828.73 ± 200.23	818.07 ± 208.91	0.825	0.411	0.274	0.784	0.595	0.553
**SBT_Accuracy**	0.86 ± 0.10	0.87 ± 0.08	0.91 ± 0.07	−3.426	**0.001**	−2.868	**0.005**	−0.740	0.461
**SBT_RT**	932.48 ± 272.44	872.02 ± 194.37	843.45 ± 194.46	2.047	**0.043**	0.774	0.441	1.386	0.169
**TMT_A**	81953.60 ± 70035.11	64174.07 ± 29571.79	61910.72 ± 31822.10	2.020	**0.047**	0.388	0.699	1.812	0.074
**TMT_B**	169321.18 ± 71840.52	178337.09 ± 200969.52	124070.86 ± 48964.28	4.009	**0.000**	1.947	0.056	−0.315	0.754

Data are presented as mean ± std; Statistical significance was set to *p* < 0.05. Abbreviations: CI_L=patients with infarct in left-sided internal capsule; CI_R=patients with infarct in right-sided internal capsule; HC=healthy controls; RAVLT_S=short-term scores of Rey Auditory Verbal Learning Test; RAVLT_L=long-term scores of Rey Auditory Verbal Learning Test; FT_Accuracy = accuracy of Flanker task; FT_RT=reaction time of Flanker task; NBT_Accuracy = accuracy of number back task; NBT_RT=reaction time of number back task; SBT_Accuracy = accuracy of spatial back task; SBT_RT=reaction time of spatial back task; TMT_A=Trail Making Test A; TMT_B=Trail Making Test B.

### 3.2 Static functional network connectivity analysis

**Fig. 4:**
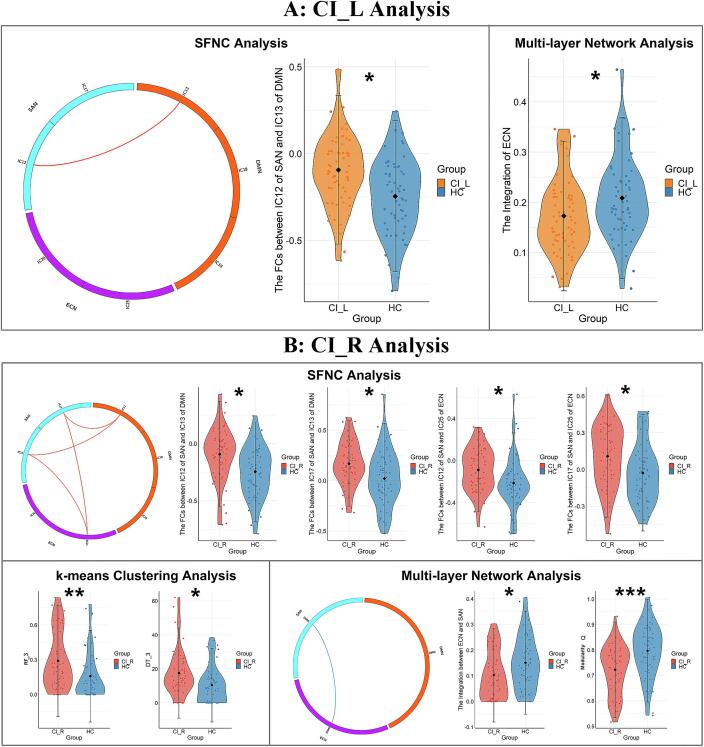
A: SFNC and DFNC results in CI_L analysis (*p* < 0.05, FDR corrected). B: SFNC and DFNC results in CI_R analysis (*p* < 0.05, FDR corrected). The red/blue lines represent increased/decreased functional connectivity/integration in stroke patients compared to HC groups. In violin plots, two horizontal lines indicate standard deviation ranges and the black points indicate the mean values. The width of the violin plot bars indicates the distribution density of subjects in each group at the corresponding ordinate level. The asterisks above violins indicate pairwise comparisons that demonstrated statistical significance: * *p_FDR_*<0.05, ** *p_FDR_*<0.01, *** *p_FDR_*<0.001. Abbreviations: DMN=default mode network; ECN = execution control network; SAN=salience network; SFNC=static functional network connectivity; HC=healthy controls; CI_L=patients with left-sided capsular stroke; CI_R=patients with right-sided capsular stroke. HC=healthy controls; ICs = independent components; RF=reoccurrence fraction; DT=dwell time.

In SFNC analysis, for the CI_L analysis (Table S1 and Fig. 4**A**), compared with HC, CI_L patients displayed increased FCs between the IC12 of SAN and IC13 of DMN (*t =* 3.469, *p_FDR_*=0.016); for the CI_R analysis (Table S1 and Fig. 4**B**), CI_R patients displayed increased FCs between the IC12 of SAN and IC13 of DMN (*t* = 3.269, *p_FDR_*=0.023), between the IC17 of SAN and IC13 of DMN (*t* = 3.147, *p_FDR_*=0.023), between the IC12 of SAN and IC25 of ECN (LECN, *t* = 2.816, *p_FDR_*=0.030), and between the IC17 of SAN and IC25 of ECN (LECN, *t* = 2.988, *p_FDR_*=0.024) compared to HC.

### 3.3 Dynamic functional network connectivity analysis

#### 3.3.1 K-means clustering analysis

In k-means clustering analysis, the DFNC matrices were clustered into four connectivity states that were recurrent throughout the resting-state fMRI acquisition and in all subjects (Fig. 3**)**. State 1, which accounted for 42 % of all windows, was characterized by extensively sparse FNCs within and between all RSNs. State 2, which accounted for 19 % of all windows, was characterized by high FNCs within and between DMN and ECN. State 3, which accounted for 22 % of all windows, was characterized by tightly positive FNCs within and between all RSNs. State 4, which accounted for 17 % of all windows, was characterized by tightly positive connections within and between DMN and IC25 of ECN (LECN). For CI_L analysis, no significant difference was found between CI_L and HC groups. For CI_R analysis, CI_R patients had an increased RF and DT in state 3 (for RF, *t* = 3.157, *p_FDR_*=0.008; for DT, *t* = 3.054, *p_FDR_*=0.011) compared to HC group. The detail information is displayed in Table S2 and Fig. 4.

#### 3.3.2 Multilayer network analysis

Neither CI_L nor CI_R differed from the HC group in terms of IC-level integration parameters (Supplementary material, Table S3). At the RSN-level, for CI_L analysis, CI_L patients displayed decreased integration of ECN compared to HC group (*t* = -2.564, *p_FDR_*=0.035; Table S4 **and** Fig. 4**A**); for CI_R analysis, CI_R patients displayed decreased integration between ECN and SAN compared to HC group (*t* = -2.599, *p_FDR_*=0.032; Table S4 **and** Fig. 4**B**). Compared to HC subjects, CI_R patients displayed decreased quality of community (modularity Q) compared to HC group (*t* = -3.856, *p_FDR_*=0.000; Table S4 **and** Fig. 4**B**), no significant difference was found in CI_L group.

### 3.4 Correlations between SFNC/DFNC parameters and clinical assessments

**Fig. 5:**
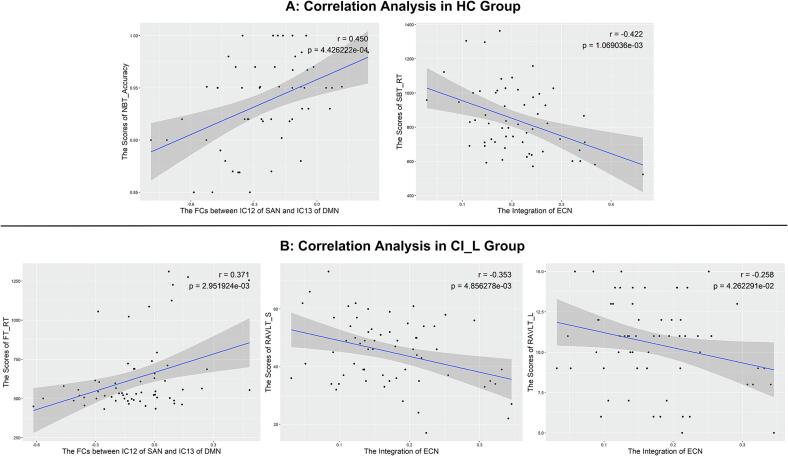
Correlation analysis in CI_L analysis. A: for HC group, the FCs between IC12 of SAN and IC13 of DMN was positively associated with the scores of NBT_Accuracy; the integration of ECN was negatively associated with the scores of SBT_RT. B: for CI_L patients, the FCs between IC12 of SAN and IC13 of DMN was positively associated with the scores of FT_RT, the integration of ECN was negatively associated with the scores of RAVLT_S and RAVLT_L. The significance level is set to *p* < 0.05.

In CI_L analysis (Fig. 5), for HC group, the FCs between IC12 of SAN and IC13 of DMN was positively associated with the working memory performance (for NBT_Accuracy: *r* = 0.450, *p* = 0.000); the integration of ECN was positively associated with spatial memory performance (for SBT_RT: *r* = -0.422, *p* = 0.001). For CI_L patients, the FCs between IC12 of SAN and IC13 of DMN was negatively associated with visual attention performance (for FT_RT*: r* = 0.371, *p* = 0.003), the integration of ECN was negatively associated with episodic memory performance (for RAVLT_S*: r* = -0.353, *p* = 0.005; for RAVLT_L: *r* = -0.258, *p* = 0.043).

**Fig. 6:**
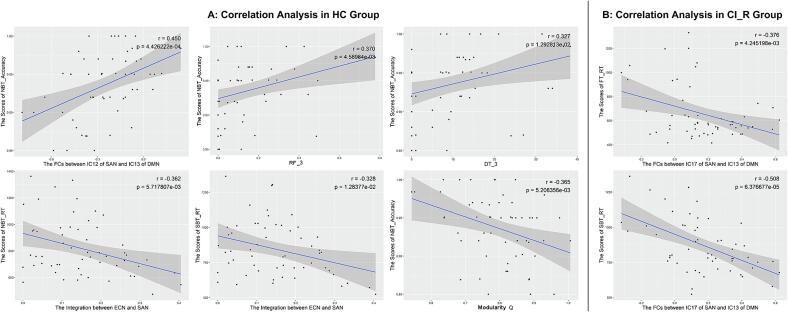
Correlation analysis in CI_R analysis. A: for HC group, the FCs between IC12 of SAN and IC13 of DMN, the RF and DT in state 3 were positively associated with the scores of NBT_Accuracy; the integration between ECN and SAN were negatively associated with the scores of NBT_RT and SBT_RT, the Q was negatively associated with the scores of NBT_Accuracy. For CI_R patients, the FCs between IC17 of SAN and IC13 of DMN was negatively associated with the scores of FT_RT and SBT_RT. The significance level is set to *p* < 0.05.

In CI_R analysis (Fig. 6), for HC group, the FCs between IC12 of SAN and IC13 of DMN (*r* = 0.450, *p* = 0.000), the RF (*r* = 0.370, *p* = 0.005) and DT (*r* = 0.327, *p* = 0.013) in state 3 were positively associated with the working memory performance; the integration between ECN and SAN were positively associated with the scores of the working memory performance (for NBT_RT: *r* = -0.362, *p* = 0.006) and spatial memory performance (for SBT_RT: *r* = -0.328, *p* = 0.013), the modularity Q was negatively associated with the working memory performance (for NBT_Accuracy: *r* = -0.365, *p* = 0.005). For CI_R patients, the FCs between IC17 of SAN and IC13 of DMN was positively associated with the visual attention performance (for FT_RT: *r* = -0.376, *p* = 0.004) and spatial memory performance (SBT_RT: *r* = -0.508, *p* = 0.000).

## 4 Discussion

The functional connectome parameters utilized in this study, encompassing SFNC, k-means clustering, and multi-layer network analyses, highlight the substantial impact of the disordered interactions across the triple-network model on the multidomain cognitive deficits observed in infarction patients. These deficits span various domains, including visual attention, episodic memory, spatial memory, working memory, and executive function. Compared to HC, CI_L and CI_R patients showed increased static FCs between SAN and DMN, and decreased dynamic interactions between ECN and other networks. CI_R patients also exhibited heightened static FCs between SAN and ECN, and maintained a state with strongly positive FNCs across all networks in the triple-network model. Additionally, CI_R patients displayed decreased modularity Q.

### 4.1 Common changes in CI_L and CI_R patients compared to HC

In SFNC analysis, CI_L and CI_R patients both displayed increased FCs between IC12 of SAN (located in the insula) and IC13 of DMN, and the FCs were negatively associated with the visual attention performance in CI_L group. The insula establishes connections with both the ECN and the DMN, contributing to the engagement of the ECN and the disengagement of the DMN in the presence of significant stimuli (Menon, 2011). According to a proposed theory (Whitfield-Gabrieli, 2009), it is believed that patients' challenges in cognitive function arise from their inability to effectively deactivate the DMN during cognitive tasks. Therefore, the heightened insula-DMN FCs observed in our study might potentially be intertwined with these pathological processes, thus contributing to the visual attention deficits in CI_L and CI_R patients. While an intriguing observation is that among the HC group, we identified a positive correlation between insula-DMN FCs and working memory performance. This finding contrasts with our discovery in the stroke group, where heightened insula-DMN FCs were associated with compromised cognitive function. We speculate that in HC group, individuals with higher cognitive flexibility also exhibit more flexible insula-DMN FCs during task execution. Even slight deactivation of DMN is sufficient to maintain a normal level of cognition. This also explains why individuals with greater working memory performance tend to have lower levels of DMN deactivation (higher insula-DMN FCs) in HC group.

In multi-layer network analysis, relative to HC, CI_L patients demonstrated diminished integration of the ECN, and CI_R patients exhibited reduced integration between the ECN and SAN. In HC group, the integration of ECN/integration between the ECN and SAN displayed a positive correlation with scores in working memory and spatial memory tests. Memory-related tasks often require both the identification of important (salient) information (Schimmelpfennig et al., 2023) and the cognitive control (Seeley, 2007) to manipulate that information, roles that SAN and ECN are thought to play, respectively. However, in CI_L patients, the level of integration of ECN exhibited an inverse correlation with episodic memory scores. This phenomenon contradicts positive correlation between integration of ECN and memory function observed in HC group. More independent operation of the ECN serves as a compensatory mechanism (Grefkes and Fink, 2011) for the potential inability of the DMN to deactivate caused by increased insula-DMN FCs in CI_L and CI_R patients. This independence helps prevent excessive interactions between the ECN and DMN, thus ensuring the normal execution of cognitive functions primarily regulated by the ECN (Fox, 2005). Consequently, the diminished dynamic interaction between ECN and other networks could paradoxically facilitate memory recovery in CI_L patients. Moreover, it was noted that in CI_R patients, the integration between ECN and SAN was also reduced. This outcome suggests that the self-reliant operating mode of ECN not only diminishes interaction with DMN but also diminishes collaboration with SAN. However, the reduced collaboration with the SAN may be a form of collateral damage, as complex cognitive tasks require the coordinated efforts of multiple networks, in which SAN is thought to identify important stimuli, while ECN is believed to implement cognitive control based on these stimuli (Seeley, 2007).

### 4.2 Distinct changes in CI_R patients compared to HC

In SFNC analysis, aside from increased FCs between IC12 (insula) of SAN and IC13 of DMN, CI_R patients also exhibit heightened FCs between the IC17 (dACC) of SAN and IC13 of DMN, and such increased dACC-DMN FCs were positively associated with the scores of visual attention and spatial memory tests, which contradicts our previous findings that FCs between the insula (also part of the SAN) and DMN are negatively correlated with scores on visual attention functions. This contrasting observation may suggest an intriguing direction: the insular cortex, which primarily functions in emotional processing, interoception, and self-awareness (Lu, 2016); appears to dilute cognitive resources when overly connected to the DMN, thereby negatively impacting cognitive functions. In contrast, the dACC, with its roles in task-switching (Kamijo, 2014), error-monitoring (Larson et al., 2013), and cognitive control (Moran et al., 2015), seems to facilitate efficient cognitive resource allocation and self-regulation when its connectivity with DMN is enhanced, may suggesting that the increase in dACC-DMN FCs is related to improvements in visual attention and spatial memory performance in CI_R patients.

In addition, consist with a previous study on stroke (Zhang, 2021), we found increased FCs between SAN and IC25 of ECN (LECN), may suggesting that the brain employs compensatory enhancement mechanisms to address cognitive deficits. Furthermore, it is noteworthy that we only observed enhanced SAN-LECN FCs in CI_R patients, without detecting alterations of SAN-RECN FCs. One possible explanation is that right hemisphere stroke may lead to impairment of the RECN, prompting the brain to compensate by strengthening connections between the LECN and other networks to offset the functional deficits of RECN (Woisard, 2023). This compensatory enhancement may represent an adaptive strategy, aimed at meeting new cognitive demands following a stroke. To further explore the issue of ECN lateralization in CI_R patients, we separately presented all the results concerning the FC status between LECN/RECN and other networks in the CI_R group versus HC (Supplementary material, Table S5). In the uncorrected condition, CI_R patients exhibited increased static FC between LECN and SAN, but showed reduced integration of RECN, as well as decreased integration between RECN and SAN. This also supports that the reduced dynamic interaction between ECN and SAN is mainly contributed by RECN. This further indicates that there is a lateralization effect of ECN in CI_R patients, where RECN tends to operate in a “more segregated” mode, while LECN leans towards a “more integrated” mode of operation.

In k-means clustering analysis, we found CI_R patients displayed increased RF and DT in state 3, characterized by tightly positive FNCs within and between all RSNs in triple-network model. In task execution, it's commonly observed that the activity of the DMN diminishes, while the activity of the ECN becomes more pronounced (Palhano-Fontes, 2015). The SAN plays a critical role in modulating this transition, acting as a mediator that helps to smoothly shift from the introspective and self-referential state to the task-execution state (Reese, 2019). Remaining in a state with enhanced FNCs among the DMN, ECN, and SAN for an extended period could have detrimental effects on cognitive flexibility, as it may limit the brain's ability to reconfigure network states when facing different tasks or environments. Additionally, we found that the RF/DT of state 3 positively correlates with scores in working memory tasks in HC group, while this relationship is absent in CI_R patients. This suggests a form of decoupling in CI_R patients between RF/DT in state 3 and cognitive activities, possibly due to a disruption in the coordination among the triple-network model.

In multi-layer network analysis, we found decreased modularity Q compared to HC group. Modularity evaluates the extent to which a network is partitioned into functionally distinct modules (communities), reflecting the brain's capacity to manage specific functions within tightly connected functional subnetwork (Paldino et al., 2017). Higher modularity Q indicates a well-organized network with clear community structures, while lower modularity Q suggests a more diffuse or overlapping community structure. Prior studies have shown that modularity is linked to various cognitive functions in healthy individuals, encompassing learning capabilities (Bassett, 2011), receptiveness to cognitive training (Gallen, 2016), and typical brain aging (Meunier et al., 2009). On the flip side, irregular modular organization has been observed in individuals with neuropsychiatric conditions, such as Alzheimer's disease (Brier, 2014), schizophrenia (Yang, 2016), traumatic brain injuries and strokes (Siegel, 2018). Multiple researches (Siegel, 2018, Tao and Rapp, 2019) have found that enhanced modularity is associated with improved post-stroke recovery in patients. In addition, dynamic interactions, quantified by the integration coefficient, provide insights into the temporal reconfiguration of these modular structures, allowing us to detect the brain's flexible adaptation to various cognitive demands in a healthy brain. By linking dynamic interactions with modularity Q, we can better understand the relationship between the temporal flexibility of brain networks, their structural organization, and cognitive outcomes in stroke patients. Our study found that CI_R patients exhibit both reduced dynamic interactions and decreased modular quality, which may indicate poor temporal flexibility and weak structural organization, potentially affecting information processing, goal-directed behavior, and cognitive control. Indeed, it is puzzling that we observed a negative correlation between the modularity of the triple-network model and working memory performance in healthy individuals. We speculate that this is because working memory tasks necessitate rapid information updating and processing (Duncan, 2013), which in turn relies on dynamic integration across different networks (Cole et al., 2014). A highly modularized network may be less efficient in tasks requiring such quick integration.

### 4.3 Heterogeneities between the CI_L and CI_R patients compared to HC

Furthermore, our study showed notable heterogeneities between the CI_L and CI_R groups compared to HC, indicating a lesion-side effect. These results align with previous findings, which revealed that patients with right hemisphere infarctions exhibited more pronounced functional activity abnormalities (Wu, 2021); greater gray matter volume (GMV) changes (Jiang, 2017), and more severe damage in the affected corticospinal tract (Diao, 2017). Several factors may contribute to this observation: The functional differences between the left and right hemispheres may play a role. The left hemisphere has a more significant role than the right hemisphere in executing movements in healthy individuals (Verstynen et al., 2005), as further confirmed in stroke patients where right hemisphere infarct patients exhibit stronger interlimb coupling compared to left hemisphere infarct patients (Lewis and Perreault, 2007). Therefore, right-sided stroke patients, whose left hemisphere remains intact, may have greater potential for brain reorganization, leading to more extensive changes in brain connectivity. The severity of brain damage is another contributing factor. Several researches have reported that right-sided stroke patients often reach hospitals later than left-sided patients, which can delay treatment and result in more severe brain damage (Di Legge et al., 2005, Di Legge et al., 2006). Generally, more severe brain damage may lead to more extensive brain reorganization, suggesting that the difference in severity of brain damage could be a key factor in the lesion-side effect on functional connectivity changes after subcortical stroke. The impact on daily activities is also significant. Right-handed patients with a right hemisphere infarction generally have fewer difficulties with daily activities compared to those with a left hemisphere infarction, reducing their engagement in daily activities conducive to recovery and potentially leading to more widespread functional connectivity changes in right hemisphere infarct patients. Additionally, lesion location differences may contribute. Slight differences in lesion locations between left and right lesion groups could also contribute to the observed lesion-side effects on brain connectivity. These factors collectively highlight the greater impact of right hemisphere strokes on brain connectivity, underscoring the importance of considering lesion side in stroke rehabilitation.

### 4.4 Limitation

Several limitations should be considered. First, the sample size was relatively small, which may limit the statistical power of the results. Second, the study employed a cross-sectional design, precluding any insights into the dynamic evolution of the disease over time. Third, the study only included right-handed participants, potentially limiting the applicability of the findings to populations with different hand dominances. Fourth, we did not perform multiple corrections on brain-behavior correlations, potentially increasing the risk of chance findings. Fifth, the lack of comprehensive information on participants' medication and treatment schedules might have introduced unconsidered confounding factors, influencing the outcomes observed. Future research should include detailed treatment protocols to offer a more thorough understanding of recovery trajectories in stroke patients. Lastly, the wide age range of 40 to 80 years could introduce variability; therefore, concentrating on narrower age groups in future studies will enable a more precise evaluation of the age-related effects on post-stroke FNC and cognitive impairments.

## 5 Conclusion

In this study, by exploring the alterations of SFNC and DFNC properties, the significant impact of disordered interactions within triple-network model on the multidomain cognitive impairments were observed in stroke patients. These intertwined changes in SFNC and DFNC within triple-network model contribute to refine the neuropathological framework for multidomain cognitive deficits in stroke patients, aiming to guide diagnostic and treatment decisions for stroke.

## Funding sources

This research did not receive any specific grant from funding agencies in the public, commercial, or not-for-profit sectors.

## CRediT authorship contribution statement

**Yingying Wang:** Writing – original draft, Visualization, Methodology, Formal analysis, Conceptualization. **Hongxu Chen:** Visualization, Validation, Software, Methodology. **Caihong Wang:** Supervision, Investigation. **Jingchun Liu:** Resources, Investigation. **Peifang Miao:** Supervision, Investigation. **Ying Wei:** Resources, Investigation. **Luobing Wu:** Supervision, Investigation. **Xin Wang:** Supervision, Investigation. **Peipei Wang:** Supervision, Investigation. **Yong Zhang:** . **Jingliang Cheng:** Project administration, Investigation, Data curation. **Siyuan Fan:** Writing – review & editing, Visualization, Software, Formal analysis, Conceptualization. **Guifang Sun:** Writing – review & editing, Validation, Supervision, Resources, Investigation, Conceptualization.

## Declaration of Competing Interest

The authors declare that they have no known competing financial interests or personal relationships that could have appeared to influence the work reported in this paper.

## Data Availability

Data will be made available on request.
