# Supplementary material for: Static and dynamic interactions within the triple-network model in stroke patients with multidomain cognitive impairments
Source: Neuroimage Clin. 2024 Aug 10;43:103655. doi: 10.1016/j.nicl.2024.103655 (PMC11367478; doi:10.1016/j.nicl.2024.103655)
Supplement: Supplementary Data 1 [file mmc1.docx]

**Part 1 The inclusion/ exclusion criteria for Stroke and HC groups**

Inclusion criteria for stroke patients were as follows: (1) first occurrence of ischemic stroke; (2) presence of a single ischemic infarct lesion involving the internal capsule; (3) right-handedness; (4) at least 6 months after stroke onset; (5) neurological deficit evident at acute onset (NIH stroke scale, NIHSS > 3); (6) age between 40 and 80 years. Exclusion criteria for patients were: (1) recurrent stroke; (2) any previous neurological or psychiatric disorders; (3) evidence of brain structure damage on MRI scans; (4) history of alcohol or drug addiction; (5) conditions preventing MRI or affecting image quality; (6) Fazekas scale score for white matter hyperintensity greater than one on T2 fluid attenuated inversion recovery (T2 FLAIR) images ^1^; (7) presence of lacunes (as observed on T2 FLAIR). The healthy controls (HC) were recruited from the local community, with the following criteria: (1) matched for sex, age, and education with CI_L and CI_R groups; (2) absence of neurological dysfunction; (3) the exclusion criteria were the same as those applied to stroke patients.

**Part 2 Behavioral Measures**

In this study, cognitive tasks were performed using E-Prime 2.0 software (https://pstnet.com/products/e-prime/). To evaluate visual attention, participants completed a version of the Flanker task (FT) ^2^ involving arrow stimuli. During this task, participants were instructed to press the left or right computer mouse button according to the direction of the central target arrow, while disregarding the surrounding "flanking" arrows. Episodic memory was estimated using the Rey Auditory Verbal Learning Test (RAVLT) ^3^. In this test, participants listened to 15 words and tried to recall them, which was repeated five times for a short-term score (RAVLT_S). After 20 minutes, they were tested again for a long-term score (RAVLT_L). Working memory and spatial memory were assessed using modified versions of the number back task (NBT) ^4^ and spatial back task (SBT) ^5^, respectively. Each task consisted of 61 trials of 1-back conditions, where participants had to memorize and recognize constantly changing visual number or spatial stimuli and compare the current stimulus information with the one immediately preceding it. Cognitive indicators were calculated based on mean reaction time (RT) for correct responses and accuracy. Executive function was gauged using the Trail Making Test (TMT) ^6^, comprising two parts (TMT-A and TMT-B). In TMT-A, participants connected numbered circles positioned randomly, following the sequence from 1 to 25. In TMT-B, they alternated between connecting circles with numbers and letters (e.g., 1 to A to 2 to B, ...). Test scores were determined by the time taken to complete these tasks. Furthermore, motor function was assessed using the Fugl-Meyer test (FMT), which encompassed tests for the whole extremity and upper limb.

**Part 3 The Detail Parameters of MR Images**

At both medical centers, MR images of all subjects were acquired using the same parameters on two scanners with the same type (Discovery MR750 3.0 Tesla, General Electric, Milwaukee, WI, USA). During the scanning, all participants were asked to close their eyes and remain motionless. A gradient echo single-shot echo-planar imaging sequence with the following parameters was used to generate resting-state fMRI data for each subject: repetition time (TR) / echo time (TE) = 2000 ms / 30 ms, flip angle = 90°, field of view (FOV) = 220 mm × 220 mm, matrix = 64 × 64, slice thickness = 4.0 mm, gap = 0.5 mm, slices = 32, and volumes=180, and total time = 6 minutes. Sagittal three-dimensional T1-weighted images (3D-T1WI) were acquired by a brain volume (BRAVO) sequence with the following parameters: TR/TE = 8.2 ms / 3.2 ms, flip angle = 12°, FOV = 256 mm × 256 mm, matrix = 256 × 256, slice thickness = 1.0 mm, no gap, slices = 188, and voxel size = 1 mm × 1 mm × 1 mm. T2 FLAIR images were acquired with the following parameters: TR/TE = 8400 ms / 155 ms, thickness = 5.0 mm, FOV = 240 mm × 240 mm, and slices = 21.

**Part 4 Data Preprocessing**

The Data Processing & Analysis for Brain Imaging (DPABI; http://rfmri.org/DPABI) program was used to preprocess all MRI data. The following procedures were performed: (a) removing the first 10 volumes; (b) slice timing; (c) realignment; (d) spatial normalization with a resampling voxel size of 3 mm × 3 mm × 3 mm, using Diffeomorphic Anatomical Registration Through Exponentiated Lie algebra (DARTEL) ^7^; (e) smoothing (6 mm × 6 mm × 6 mm full-width at half maximum Gaussian kernel).

**Part 5 Controlling for Head Motion**

To reduce the potential impacts of head motion on SFNC and DFNC, we took the following actions. To begin, we excluded participants who had a maximum displacement of more than 2.0 mm and a maximum rotation of more than 2.0 degrees. Second, we calculated mean framewise displacement (FD) ^8^ by averaging each participant's FD across time points for removing participants with mean FD > 0.5, and there was no significant difference of mean FD between the CI_L and the HC groups (*p* = 0.056), between the CI_R and the HC groups (*p* = 0.081), as well as between the CI_L and the CI_R groups (*p* = 0.977). Thirdly, using ICA, which has been demonstrated to reliably eliminate motion-related noise from fMRI data, the "artifactual" components were identified and deleted ^9^. Fourthly, despiking was done on the IC time-courses that were chosen, the data points with a root mean square of the frame-wise displacement > 0.5 mm were identified as spikes by using 3Ddespike algorithm implemented in Analysis of Functional NeuroImages (AFNI) ^10^, adjusted utilizing a third-order spline fit to the clean sections of the input ^11^. This approach is similar to the “scrubbing” method ^12^, but it avoids removing volumes that would disturb the temporal continuity necessary ^13^ for SFNC and DFNC analyses. According to previous research ^14^, despiking could reduce the influence of outliers on FNC studies. In addition, using six realignment parameters, motion was regressed out of the data during DFNC processing. These strategies work together to deliver a powerful combination of linear and non-linear motion reduction.

Part 6 Multilayer Network Analysis of DFNC

1 Multilayer Network Construction

While we divided the total time series into 148 windows for each subject, it's important to note that single graphs within the time-evolving networks hold some reliance on the graphs constructed from previous and subsequent time windows. To address this, we adopted a multilayer network approach to account for this temporal interdependence among windows. In this approach, an adjacency matrix was estimated for each time window, and all 148 windows were interconnected into a multilayer network. Within this framework, every independent component (IC) was represented as a node, Pearson's correlations between each pair of ICs were depicted as edges, and each time window corresponded to a layer. We exclusively considered connectivities between the same ICs across adjacent layers. A multilayer network can be simplified through a supra-adjacency matrix ^15^. For an f-layer multilayer network, its supra-adjacency matrix is described as follows:


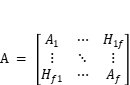


where *A_α_* denotes the adjacency matrix of the intralayer network in layer *α* (*1 ≤ α ≤ f)*. *H_kl_* corresponds to the interlayer connection matrix connecting layer *k* to layer *l*.

**2 Multilayer Community Detection**

To explore the temporal evolution of community in the multilayer network, we employed the GenLouvain community detection algorithm ^16^, rooted in the concept of the Louvain-like greedy algorithm. Modularity serves as a formal metric for investigating the optimal assignment of these communities. In essence, this algorithm determines the community membership for each IC by maximizing the multilayer modularity quality function Q, which is defined as:


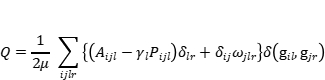


where *µ* stands for the total edge weight in the network, *A_ijl_* represents the correlation matrix between ICs *i* and *j* for layer *l*, and *P_ijl_* signifies the expected correlation in the Newman–Girvan null model. The parameter *γ_l_* refers to the topological resolution parameter of layer *l*, related to the edge weight of intralayer network; whereas *ω_jlr_* is the temporal coupling parameter that sets the edge weight of interlayer network. Since there is no recognized standard method for determining these parameters, in this study, we set *γ* = *ω* = 1 as was done in prior work ^17^. The quantities *g_il_* and *g_jr_* represent the community assignments of IC *i* in layer *l* and IC *j* in layer *r* respectively. The use of the Kronecker delta function, *δ*, results in *δ(g_il_, g_jr_)* being equal to 1 when *g_il_ = g_jr_*, and otherwise equal to 0. If *l* = *r*, *δ_lr_* = 1 and equals 0 otherwise; if and only if *i* = *j*, *δ_ij_* = 1. For each time window (layer), we derived IC community partitions by optimizing the multilayer modularity function Q. Due to the inherent randomness of the Louvain-like greed algorithm, the output varies slightly across runs ^18^. Therefore, we executed this algorithm 50 times to obtain final average values for quantitative indicators (integration coefficient) in each run.

**3 Multilayer Network Properties Analysis**

**Module Allegiance**

Module allegiance characterizes the consistency of community assignment between two ICs during the scan. An N × N square matrix (N is the number of ICs) was formed to create module allegiance matrix for each participant, in which each element *P_ij_* gives the relative frequency that one IC shares the same community with another IC across time windows ^19^. It can be represented as follows:


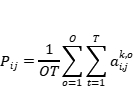


where *O* represents the repetition times of the multilayer community detection algorithm, *T* represents the number of layers, and element *a^k,o^ _i,j_* = 1 if ICs *i* and *j* are in the same community and 0 otherwise. Correspondingly, the *P_ij_* = 1 if ICs *i* and *j* are always in the same community and 0 if they are never in the same community. The module allegiance between two RSNs is also calculated by averaging the *P_ij_* of all ICs between the two RSNs.

**Integration**

The integration coefficient ^20^, calculated from the module allegiance matrix, quantifies the dynamic interactions between ICs within a network and ICs from other networks ^15^. These interactions involve reconfiguration over time, where ICs in the same community at one time point may shift to join communities with ICs from other networks at the next time point. The higher the integration coefficient of an IC, the easier it is to communicate with ICs in other networks across time windows. For an IC *i* in functional network *S*, the integration (*I*) can be defined as:


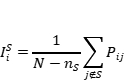


where *N* is the total number of ICs, *n_s_* is the number of ICs in network *S*. The IC-level integration was averaged across each RSN to obtain the RSN-level integration coefficient. The RSN-RSN integration is represented by the module allegiance matrix between RSNs.

Part 7 Scanning Duration Considerations

We selected a 6-minute (180 time-points) scanning duration to investigate the community structure of dynamic brain networks, a topic that remains highly debated within the field of dynamic neuroscience. The choice of this duration is based on previous studies that have shown such a time frame can successfully capture meaningful dynamic changes in brain networks ^11,21^. For instance, research by Tu et al. and Mennigen et al. indicates that a 6-minute scan is sufficient to observe significant patterns of change in dynamic functional network connectivity of the brain in patients with migraine and schizophrenia. Even shorter scan durations have been used to examine dynamic changes in patients with mild brain injuries ^22^ and Alzheimer's disease ^23^. This scanning length was chosen to balance practical feasibility with the need to acquire a sufficient number of data points to ensure reliable computation of network metrics over time. Practical limitations, such as the availability of scanning equipment, participant comfort, and cost considerations, often restrict the feasible duration of neuroimaging experiments, making longer scans challenging. However, we acknowledge that a 6-minute duration may not capture all potential dynamic variations within brain networks, particularly slower or more subtle changes that could exceed this time window. Therefore, we emphasize that future research should experiment with different scanning durations to further explore brain dynamics.

**Table S1** Inter-network connectivity (SFNC) differences between stroke patients and healthy controls.

| **Variables** | **Mean ± STD** | | | **CI_L vs HC** | | **CI_R vs HC** | |
| --- | --- | --- | --- | --- | --- | --- | --- |
|  | **CI_L** | **CI_R** | **HC** | ***t*** | ***p_FDR_*** | ***t*** | ***p_FDR_*** |
| **SAN12-DMN13** | -0.09±0.21 | -0.09±0.23 | -0.25±0.22 | 3.469 | **0.016** | 3.269 | **0.023** |
| **SAN17-DMN13** | 0.15±0.25 | 0.17±0.21 | 0.02±0.27 | 2.368 | 0.180 | 3.147 | **0.023** |
| **SAN12-ECN25** | -0.11±0.21 | -0.09±0.20 | -0.21±0.24 | 2.261 | 0.180 | 2.816 | **0.030** |
| **SAN17-ECN25** | 0.04±0.23 | 0.11±0.25 | -0.03±0.24 | 1.364 | 0.416 | 2.988 | **0.024** |

Data are presented as mean ± std. The significance level is set as: *p* < 0.05, FDR corrected. Abbreviations: CI_L = patients with infarct in left-sided internal capsule; CI_R = patients with infarct in right-sided internal capsule; HC = healthy controls; SAN = salience network; DMN = default mode network; ECN = execution control network.

**Table S2** The temporal properties for each state and each group.

| **Variables** | **Mean ± STD** | | | **CI_L vs HC** | | **CI_R vs HC** | |
| --- | --- | --- | --- | --- | --- | --- | --- |
|  | **CI_L** | **CI_R** | **HC** | ***t*** | ***p_FDR_*** | ***t*** | ***p_FDR_*** |
| **RF_1** | 0.48±0.27 | 0.39±0.27 | 0.38±0.30 | 1.506 | 0.251 | -0.325 | 0.746 |
| **RF_2** | 0.17±0.20 | 0.18±0.18 | 0.24±0.23 | -1.323 | 0.251 | -1.116 | 0.356 |
| **RF_3** | 0.21±0.20 | 0.29±0.24 | 0.16±0.20 | 1.072 | 0.286 | 3.157 | **0.008** |
| **RF_4** | 0.14±0.17 | 0.13±0.15 | 0.22±0.21 | -1.933 | 0.223 | -2.087 | 0.079 |
| **DT_1** | 28.32±25.06 | 23.29±19.78 | 26.25±31.96 | 0.093 | 0.926 | -1.054 | 0.294 |
| **DT_2** | 11.71±11.64 | 11.32±10.15 | 15.52±14.63 | -1.256 | 0.423 | -1.443 | 0.203 |
| **DT_3** | 13.93±13.04 | 17.54±13.27 | 10.47±10.87 | 1.423 | 0.423 | 3.054 | **0.011** |
| **DT_4** | 11.40±13.09 | 8.48±7.00 | 14.75±17.19 | -0.900 | 0.494 | -1.994 | 0.097 |
| **TTN** | 6.45±2.60 | 7.18±2.66 | 6.63±3.17 | -0.012 | 0.990 | 1.416 | 0.160 |

Data are presented as mean ± std. The significance level is set as: *p* < 0.05, FDR corrected. Abbreviations: RF = reoccurrence fraction; DT = mean dwell time. TTN = total transition number.

**Table S3** The IC-level parameters in stroke patients and healthy controls in multi-layer network analysis.

| **Variables** | **Mean ± Std** | | | **CI_L vs. HC** | | **CI_R vs. HC** | |
| --- | --- | --- | --- | --- | --- | --- | --- |
|  | **CI_L** | **CI_R** | **HC** | ***t*** | ***p_FDR_*** | ***t*** | ***p_FDR_*** |
| Integration of IC13 of DMN | 0.149±0.09 | 0.151±0.073 | 0.158±0.073 | -0.786 | 0.623 | -0.481 | 0.632 |
| Integration of IC18 of DMN | 0.216±0.09 | 0.202±0.107 | 0.212±0.079 | 0.059 | 0.953 | -0.797 | 0.499 |
| Integration of IC19 of DMN | 0.133±0.089 | 0.139±0.071 | 0.153±0.074 | -1.202 | 0.541 | -0.866 | 0.499 |
| Integration of IC25 of ECN | 0.194±0.094 | 0.209±0.1 | 0.24±0.115 | -2.376 | 0.134 | -1.309 | 0.338 |
| Integration of IC26 of ECN | 0.152±0.075 | 0.145±0.07 | 0.177±0.071 | -1.947 | 0.189 | -2.358 | 0.141 |
| Integration of IC12 of SAN | 0.111±0.065 | 0.103±0.065 | 0.117±0.057 | -0.766 | 0.623 | -1.750 | 0.194 |
| Integration of IC17 of SAN | 0.123±0.075 | 0.101±0.058 | 0.125±0.069 | -0.162 | 0.953 | -1.924 | 0.194 |

Data are presented as mean ± std. The significance level is set as: *p* < 0.05, FDR

corrected.

**Table S4** The multi-layer network parameters in stroke patients and healthy controls.

| **Variables** | **Mean ± STD** | | | **CI_L vs. HC** | | **CI_R vs. HC** | |
| --- | --- | --- | --- | --- | --- | --- | --- |
|  | **CI_L** | **CI_R** | **HC** | ***t*** | ***p_FDR_*** | ***t*** | ***p_FDR_*** |
| **Integration of DMN** | 0.166±0.072 | 0.164±0.071 | 0.174±0.056 | -0.811 | 0.607 | -0.896 | 0.372 |
| **Integration of ECN** | 0.173±0.074 | 0.177±0.072 | 0.208±0.08 | -2.564 | **0.035** | -2.027 | 0.068 |
| **Integration of SAN** | 0.117±0.06 | 0.102±0.058 | 0.121±0.052 | -0.516 | 0.607 | -2.082 | 0.068 |
| **Integration between DMN and ECN** | 0.212±0.108 | 0.227±0.104 | 0.247±0.112 | -1.759 | 0.122 | -0.850 | 0.596 |
| **Integration between DMN and SAN** | 0.119±0.079 | 0.102±0.079 | 0.102±0.066 | 1.153 | 0.252 | -0.319 | 0.751 |
| **Integration between ECN and SAN** | 0.113±0.09 | 0.103±0.091 | 0.151±0.1 | -2.099 | 0.114 | -2.599 | **0.032** |
| **Global_level Integration** | 0.154±0.059 | 0.15±0.058 | 0.169±0.048 | -1.595 | 0.113 | -1.900 | 0.060 |
| **Q** | 0.772±0.096 | 0.722±0.093 | 0.797±0.1 | -1.291 | 0.199 | -3.856 | **0.000** |

Data are presented as mean ± std. The significance level is set as: *p* < 0.05, FDR corrected.

**Table S5** LECN/RECN-related parameters in CI_R patients compared to healthy controls

| **Variables** | **Mean ± Std** | | **CI_R vs. HC** | | |
| --- | --- | --- | --- | --- | --- |
|  | **CI_R** | **HC** | ***t*** | ***p*** | ***p_FDR_*** |
| The FCs between LECN and IC13 of DMN | 0.47±0.28 | 0.5±0.28 | -0.439 | 0.662 | 0.817 |
| The FCs between LECN and IC18 of DMN | 0.46±0.25 | 0.46±0.29 | 0.459 | 0.647 | 0.817 |
| The FCs between LECN and IC19 of DMN | 0.39±0.28 | 0.43±0.26 | -0.124 | 0.902 | 0.947 |
| The FCs between LECN and IC12 of SAN | -0.09±0.2 | -0.21±0.24 | 2.816 | **0.006** | **0.030** |
| The FCs between LECN and IC17 of SAN | 0.11±0.25 | -0.03±0.24 | 2.988 | **0.003** | **0.024** |
| The Integration of LECN | 0.209±0.1 | 0.24±0.115 | -1.309 | 0.193 | 0.338 |
| The Integration between LECN and IC13 of DMN | 0.226±0.204 | 0.217±0.232 | 0.556 | 0.579 | 0.865 |
| The Integration between LECN and IC18 of DMN | 0.083±0.12 | 0.079±0.114 | -0.162 | 0.872 | 0.992 |
| The Integration between LECN and IC19 of DMN | 0.256±0.267 | 0.246±0.234 | -0.096 | 0.924 | 0.992 |
| The Integration between LECN and IC12 of SAN | 0.217±0.231 | 0.24±0.237 | -0.501 | 0.617 | 0.865 |
| The Integration between LECN and IC17 of SAN | 0.038±0.08 | 0.03±0.065 | 0.242 | 0.809 | 0.992 |
| The FCs between RECN and IC13 of DMN | 0.31±0.22 | 0.25±0.24 | 1.339 | 0.183 | 0.385 |
| The FCs between RECN and IC18 of DMN | 0.23±0.24 | 0.16±0.24 | 0.981 | 0.329 | 0.500 |
| The FCs between RECN and IC19 of DMN | 0.16±0.24 | 0.12±0.22 | 0.972 | 0.333 | 0.500 |
| The FCs between RECN and IC12 of SAN | 0.08±0.23 | 0.06±0.23 | -0.342 | 0.733 | 0.855 |
| The FCs between RECN and IC17 of SAN | 0.15±0.27 | 0.09±0.26 | 0.783 | 0.436 | 0.610 |
| The Integration of RECN | 0.145±0.07 | 0.177±0.071 | -2.358 | **0.020** | 0.141 |
| The Integration between RECN and IC13 of DMN | 0.341±0.264 | 0.397±0.289 | -1.069 | 0.287 | 0.865 |
| The Integration between RECN and IC18 of DMN | 0.143±0.166 | 0.146±0.159 | -0.011 | 0.991 | 0.992 |
| The Integration between RECN and IC19 of DMN | 0.022±0.05 | 0.025±0.056 | -1.001 | 0.319 | 0.865 |
| The Integration between RECN and IC12 of SAN | 0.059±0.1 | 0.071±0.151 | -0.518 | 0.605 | 0.865 |
| The Integration between RECN and IC17 of SAN | 0.136±0.176 | 0.225±0.207 | -2.347 | **0.021** | 0.218 |

Data are presented as mean ± std. The significance level is set as: *p* < 0.05.


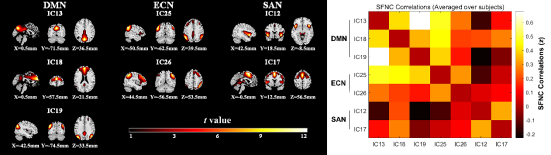
**Figure S1:** Left: The RSNs extracted by ICA. The color scale represents the *t* values in each RSN. Right: Static functional network connectivity between ICs resulting in a total of 21 connectivity pairs. Connectivity values correspond to the Fisher’s *z*-transformed Pearson correlation, averaged over the entire group of healthy controls and infarct patients. Abbreviations: DMN = default mode network; ECN = execution control network; SAN = salience network; ICs = independent components.


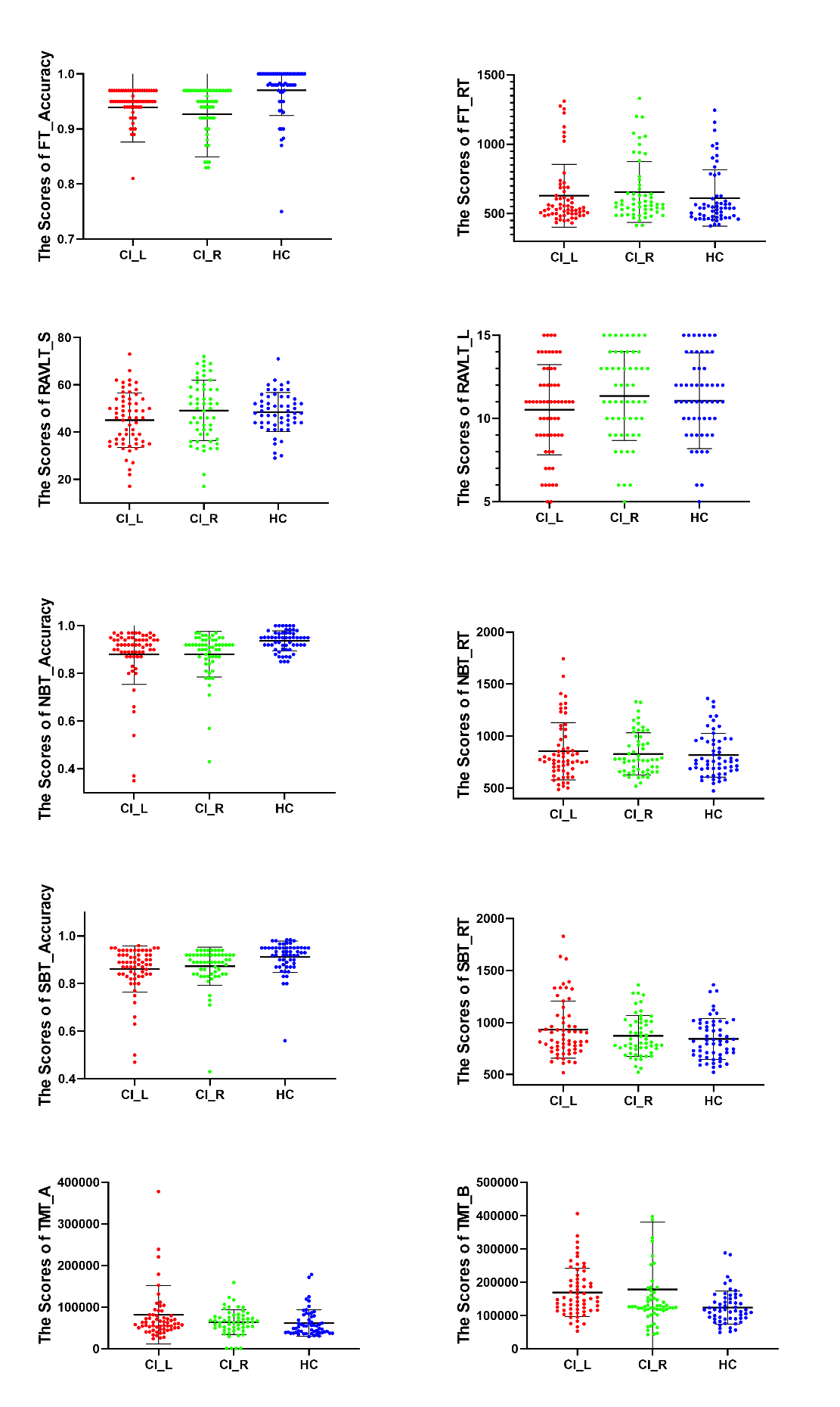


**Figure S2:** The distribution of scores from the cognitive function assessments. Abbreviations: CI_L = patients with infarct in left-sided internal capsule; CI_R = patients with infarct in right-sided internal capsule; HC = healthy controls; RAVLT_S = short-term scores of Rey Auditory Verbal Learning Test; RAVLT_L = long-term scores of Rey Auditory Verbal Learning Test; FT_Accuracy = accuracy of Flanker task; FT_RT = reaction time of Flanker task; NBT_Accuracy = accuracy of number back task; NBT_RT = reaction time of number back task; SBT_ Accuracy = accuracy of spatial back task; SBT_RT = reaction time of spatial back task; TMT_A = Trail Making Test A; TMT_B = Trail Making Test B.

**REFERENCES**

1 Fazekas, F., Chawluk, J. B., Alavi, A., Hurtig, H. I. & Zimmerman, R. A. MR signal abnormalities at 1.5 T in Alzheimer's dementia and normal aging. *AJR Am J Roentgenol* **149**, 351-356, doi:10.2214/ajr.149.2.351 (1987).

2 ERIKSEN, B. A. & ERIKSEN, C. W. Effects of noise letters upon the identification of a target letter in a nonsearch task. *Perception &Psychophysics* **16**, 143-149 (1974).

3 Allen, P. *et al.* Chiari 1000 Registry Project: assessment of surgical outcome on self-focused attention, pain, and delayed recall. *Psychological medicine* **48**, 1634-1643, doi:10.1017/s0033291717003117 (2018).

4 Stollstorff, M. *et al.* Neural response to working memory load varies by dopamine transporter genotype in children. *NeuroImage* **53**, 970-977, doi:10.1016/j.neuroimage.2009.12.104 (2010).

5 Vuontela, V. *et al.* Audiospatial and visuospatial working memory in 6-13 year old school children. *Learning & memory (Cold Spring Harbor, N.Y.)* **10**, 74-81, doi:10.1101/lm.53503 (2003).

6 Llinàs-Reglà, J. *et al.* The Trail Making Test. *Assessment* **24**, 183-196, doi:10.1177/1073191115602552 (2017).

7 Ashburner, J. A fast diffeomorphic image registration algorithm. *Neuroimage* **38**, 95-113, doi:10.1016/j.neuroimage.2007.07.007 (2007).

8 Power, J., Barnes, K., Snyder, A., Schlaggar, B. & Petersen, S. Spurious but systematic correlations in functional connectivity MRI networks arise from subject motion. *NeuroImage* **59**, 2142-2154, doi:10.1016/j.neuroimage.2011.10.018 (2012).

9 Uddin, L. Mixed Signals: On Separating Brain Signal from Noise. *Trends in cognitive sciences* **21**, 405-406, doi:10.1016/j.tics.2017.04.002 (2017).

10 Cox, R. AFNI: software for analysis and visualization of functional magnetic resonance neuroimages. *Computers and biomedical research, an international journal* **29**, 162-173, doi:10.1006/cbmr.1996.0014 (1996).

11 Mennigen, E. *et al.* Transient Patterns of Functional Dysconnectivity in Clinical High Risk and Early Illness Schizophrenia Individuals Compared with Healthy Controls. *Brain Connect* **9**, 60-76, doi:10.1089/brain.2018.0579 (2019).

12 Power, J. D., Barnes, K. A., Snyder, A. Z., Schlaggar, B. L. & Petersen, S. E. Spurious but systematic correlations in functional connectivity MRI networks arise from subject motion. *Neuroimage* **59**, 2142-2154, doi:10.1016/j.neuroimage.2011.10.018 (2012).

13 Nomi, J. S. *et al.* Chronnectomic patterns and neural flexibility underlie executive function. *Neuroimage* **147**, 861-871, doi:10.1016/j.neuroimage.2016.10.026 (2017).

14 Allen, E. A. *et al.* Tracking whole-brain connectivity dynamics in the resting state. *Cereb Cortex* **24**, 663-676, doi:10.1093/cercor/bhs352 (2014).

15 Cozzo, E. & Moreno, Y. Characterization of multiple topological scales in multiplex networks through supra-Laplacian eigengaps. *Physical review. E* **94**, 052318, doi:10.1103/PhysRevE.94.052318 (2016).

16 Jutla, I. S., Jeub, L. G. S. & Mucha, P. J. A generalized Louvain method for community detection implemented in MATLAB. <http://netwiki.amath.unc.edu/GenLouvain>. (2011).

17 He, L. *et al.* Brain flexibility associated with need for cognition contributes to creative achievement. *Psychophysiology* **56**, e13464, doi:10.1111/psyp.13464 (2019).

18 Betzel, R. F., Satterthwaite, T. D., Gold, J. I. & Bassett, D. S. Positive affect, surprise, and fatigue are correlates of network flexibility. *Sci Rep* **7**, 520, doi:10.1038/s41598-017-00425-z (2017).

19 Chai, L. R., Mattar, M. G., Blank, I. A., Fedorenko, E. & Bassett, D. S. Functional Network Dynamics of the Language System. *Cereb Cortex* **26**, 4148-4159, doi:10.1093/cercor/bhw238 (2016).

20 Cui, X. *et al.* Analysis of Dynamic Network Reconfiguration in Adults with Attention-Deficit/Hyperactivity Disorder Based Multilayer Network. *Cereb Cortex* **31**, 4945-4957, doi:10.1093/cercor/bhab133 (2021).

21 Tu, Y. *et al.* Abnormal thalamocortical network dynamics in migraine. *Neurology* **92**, e2706-e2716, doi:10.1212/WNL.0000000000007607 (2019).

22 Hou, W. *et al.* Dynamic Functional Network Analysis in Mild Traumatic Brain Injury. *Brain Connect* **9**, 475-487, doi:10.1089/brain.2018.0629 (2019).

23 Fu, Z. *et al.* Altered static and dynamic functional network connectivity in Alzheimer's disease and subcortical ischemic vascular disease: shared and specific brain connectivity abnormalities. *Hum Brain Mapp* **40**, 3203-3221, doi:10.1002/hbm.24591 (2019).
